# Room-Temperature (RT) Extended Short-Wave Infrared (e-SWIR) Avalanche Photodiode (APD) with a 2.6 µm Cutoff Wavelength

**DOI:** 10.3390/mi15080941

**Published:** 2024-07-24

**Authors:** Michael Benker, Guiru Gu, Alexander Z. Senckowski, Boyang Xiang, Charles H. Dwyer, Robert J. Adams, Yuanchang Xie, Ramaswamy Nagarajan, Yifei Li, Xuejun Lu

**Affiliations:** 1 Department of Electrical and Computer Engineering, University of Massachusetts Dartmouth, North Dartmouth, MA 02747, USA; mbenker@umassd.edu (M.B.); yifei.li@umassd.edu (Y.L.); 2 Department of Physics, Stonehill College, Easton, MA 02357, USA; ggu@stonehill.edu (G.G.); cdwyer@stonehill.edu (C.H.D.); radams3@stonehill.edu (R.J.A.); 3 Harnessing Emerging Research Opportunities to Empower Soldiers (HEROES), University of Massachusetts Lowell, One University Avenue, Lowell, MA 01854, USA; alexander_senckowski@uml.edu; 4 Department of Civil and Environmental Engineering, University of Massachusetts Lowell, One University Avenue, Lowell, MA 01854, USA; boyang_xiang@uml.edu (B.X.); yuanchang_xie@uml.edu (Y.X.); 5 Department of Plastic Engineering, University of Massachusetts Lowell, One University Avenue, Lowell, MA 01854, USA; ramaswamy_nagarajan@uml.edu; 6 USARMY DEVCOM SC, 10 General Greene Ave, Natick, MA 01760, USA

**Keywords:** extended shortwave infrared (e-SWIR), avalanche photodiodes (APDs), room temperature (RT) operation, standoff chemical sensing, infrared (IR) imaging

## Abstract

Highly sensitive infrared photodetectors are needed in numerous sensing and imaging applications. In this paper, we report on extended short-wave infrared (e-SWIR) avalanche photodiodes (APDs) capable of operating at room temperature (RT). To extend the detection wavelength, the e-SWIR APD utilizes a higher indium (In) composition, specifically In_0.3_Ga_0.7_As_0.25_Sb_0.75_/GaSb heterostructures. The detection cut-off wavelength is successfully extended to 2.6 µm at RT, as verified by the Fourier Transform Infrared Spectrometer (FTIR) detection spectrum measurement at RT. The In_0.3_Ga_0.7_As_0.25_Sb_0.75_/GaSb heterostructures are lattice-matched to GaSb substrates, ensuring high material quality. The noise current at RT is analyzed and found to be the shot noise-limited at RT. The e-SWIR APD achieves a high multiplication gain of M~190 at a low bias of $V_{bias}=-\;2.5\;V$ under illumination of a distributed feedback laser (DFB) with an emission wavelength of 2.3 µm. A high photoresponsivity of R>140 A/W is also achieved at the low bias of $V_{bias}=-2.5\;V$. This type of highly sensitive e-SWIR APD, with a high internal gain capable of RT operation, provides enabling technology for e-SWIR sensing and imaging while significantly reducing size, weight, and power consumption (SWaP).

## 1. Introduction

Extended shortwave infrared (e-SWIR) APDs, covering the wavelength range from 900 nm to 3000 nm, can provide internal gains through the avalanche multiplication of photoexcited carriers (electrons and holes). This enables the detection of weak e-SWIR signals and, thus, can find many applications in highly sensitive photodetection, standoff chemical sensing, and infrared (IR) imaging [1,2,3,4,5,6].

Various e-SWIR APD materials have been developed, including In_x_Ga_1−x_As on InP substrate with a higher indium (In) composition [7,8,9,10,11]. Indium arsenide (InAs)/GaSb type-II superlattices [12,13,14,15,16], AlInAsSb/GaSb [17], InGaAsSb lattice-matched on GaSb nBn [18], unipolar barrier e-SWIR photodetectors [19], colloidal III–V quantum dot (QD) [20], germanium–tin (Ge_1−x_Sn_x_) alloys with tunable Sn composition x [21,22,23,24], and germanium–lead (Ge_1−x_Pb_x_) alloys are used [25]. E-SWIR FPA technologies have also been developed [26,27,28]. For e-SWIR APD FPA applications, it is highly desirable to have low bias voltages of $V_{bias}<-5\;V$ to simplify the driving circuits for APDs. The breakdown voltages of APDs depend on both the bandgap $E_{g}$ of the APD semiconductor material and the device structures. APDs with a low breakdown voltage of <−1.6 V have been demonstrated using a stepwise homojunction design [29].

In this paper, we report on a new e-SWIR APD based on the In_0.3_Ga_0.7_As_0.25_Sb_0.7 5_/GaSb heterostructures lattice-matched to the GaSb subsrate. The lattice-matched heterostructure on the substrate offers high-quality materials with low strain-induced defects for low dark current. By increasing the indium (In) compostion to 0.3, the e-SWIR APD achieves a long detection cutoff wavelength of $\lambda_{cutoff}=2.6\;\mu m$. The new In_0.3_Ga_0.7_As_0.25_Sb_0.75_/GaSb heterostructure e-SWIR APD also features a separated absorption and multiplication (SAM) structure [30] to reduce the excess noise factor. By engineering the charge field of the In_0.3_Ga_0.7_As_0.25_Sb_0.75_/GaSb heterostructures, we also demonstrate a high multiplication gain of M~190 at a low bias of $V_{bias}=-2.5\;V$ under the illumination of a distributed feedback laser (DFB) with an emission wavelength of 2.3 µm. The APD shows an excess noise factor of F~500, corresponding to a k-factor of k~0.003. Table 1 summarizes the comparison of this e-SWIR APD with previously reported APDs.

## 2. Device Structures, Material Growth, and the Device Fabrication

**Figure 1:**
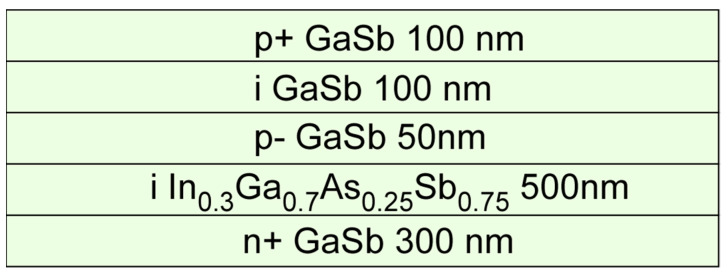
Layer-by-layer structure of the In_0.3_Ga_0.7_As_0.25_Sb_0.75_/GaSb e-SWIR APD.

Figure 1 shows a cross-sectional, layer-by-layer diagram of the new In_0.3_Ga_0.7_As_0.25_Sb_0.75_/GaSb heterostructure e-SWIR APD. It consists of, from bottom (i.e., GaSb substrate) to top, *n* ($5.0\times 10^{18}/{cm}^{3}$) tellurium (Te)-doped GaSb substrate, a 300 nanometer (nm) thick *n +* ($2.0\times 10^{19}/{cm}^{3}$) Te-doped contacting layer, an undoped 500 nm In_0.3_Ga_0.7_As_0.25_Sb_0.75_ active absorption layer, a *p*- $\left(1.5\times 10^{17}/{cm}^{3}\right)$ beryllium (Be)-doped GaSb layer, an undoped GaSb layer (i-GaSb) as the avalanche region, and the *p* + $\left(3.0\times 10^{18}/{cm}^{3}\right)$ GaSb top contacting layer. The thickness of each layer is marked in the figure. The wider bandgap i-GaSb region was designed as the avalanche region to reduce dark current- and carrier generation/recombination (GR)-induced noise, thereby lowering both dark current and noise levels during the avalanche process [17,30,31].

**Figure 2:**
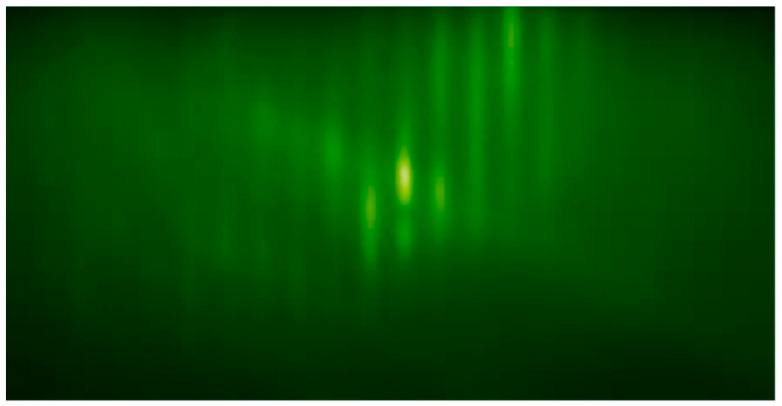
RHEED pattern after the growth was finished.

The In_0.3_Ga_0.7_As_0.25_Sb_0.75_/GaSb e-SWIR APD heterostructure was grown using a Veeco GEN Xplor Molecular Beam Epitaxy (MBE) at Tufts University Epitaxy Core facility (TEC). The GaSb wafer was ramped up to a substrate temperature of 620 °C and kept at this temperature for 10 min to remove the native oxide layer. After the de-oxidization, the substrate temperature was reduced to 542 °C. The growth temperature was kept at 542 °C throughout the growth of the material. The growth rate for the In_0.3_Ga_0.7_As_0.25_Sb_0.75_ layer was 0.74 monolayers (ML) per second (i.e., 0.74 ML/s), and the growth rate for the GaSb layers was 0.52 ML/s. The growth rates were calibriated by reflection high-energy electron diffraction (RHEED) oscillation on the MBE machine right before the MBE growth, which ensured the accuracy of the layer thicknesses shown in Figure 1. Figure 2 shows the RHEED pattern after finishing the growth. The RHEED indicates high-quality lattice-matched In_0.3_Ga_0.7_As_0.25_Sb_0.75_/GaSb heterostructure growth on the GaSb substrate. The RHEED paterns were monitored throughout the material’s growth period to ensure lattice-matching was achieved for all the layers.

**Figure 3:**
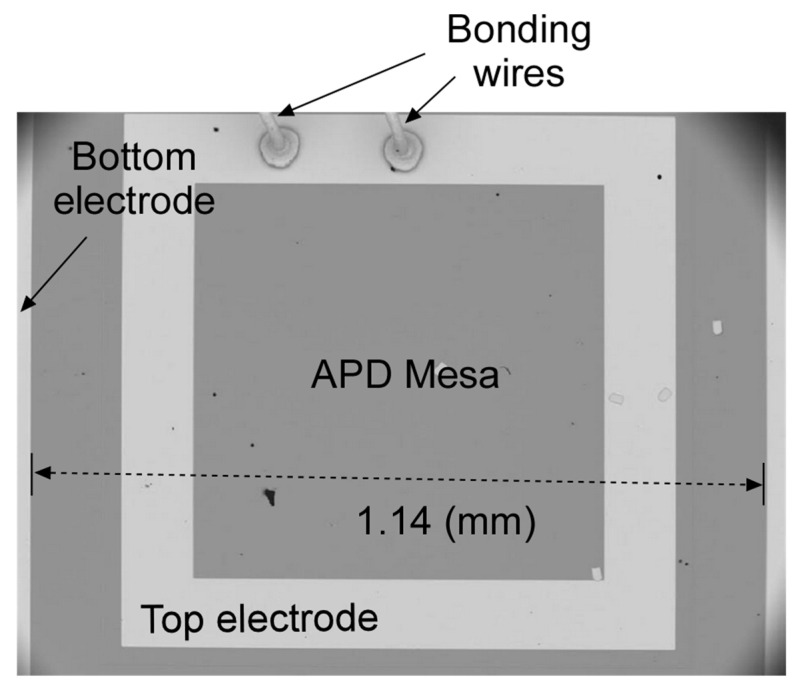
SEM image of the e-SWIR APD with bonding wires on the top electrode.

After the MBE growth, the wafer was processed into 1.1 mm × 1.1 mm square mesas using standard photolithography, inductively coupled plasma (ICP) etching, electron-beam (E-beam) metal deposition, and lift-off procedures. The ICP etching parameters were: BCl_3_, 10 standard cubic centimeters per minute (sccm), H_2_ 5 sccm, 500 W ICP, 100 W RF, and a pressure of 2 millitorrs. The total etch depth was 850 nm, with a total etch time of 9 min 30 s. The top and bottom contacts were 20 nm titanium (Ti) and 300 nm gold (Au), deposited using the E-beam metal deposition and lift-off processes. Figure 3 shows a scanning electron microscope (SEM) image of the fabricated e-SWIR APD with a square mesa and bonding wires on the top electrode.

## 3. Results and Discussions

**Figure 4:**
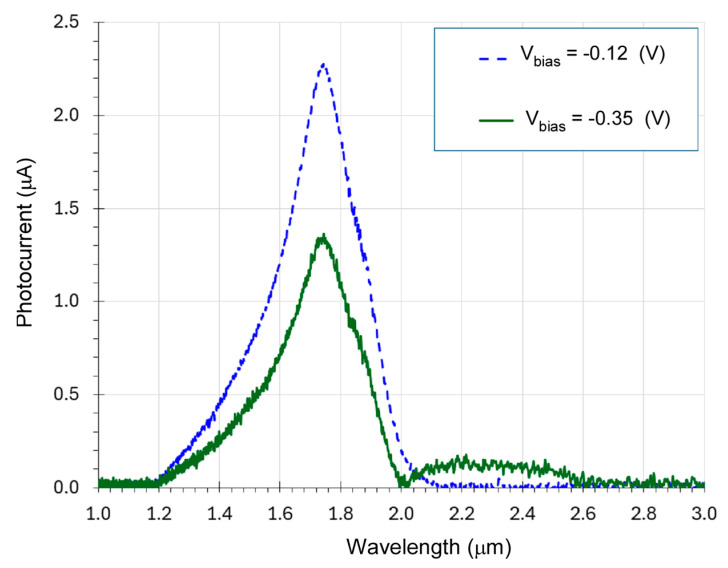
Measured FTIR photocurrent spectra of the APD at different reverse biases. At $V_{bias}=-0.12$ (V) (dashed curve), the photocurrent spectrum was mainly below the cutoff wavelength of 2.0 µm, whereas at a higher bias of $V_{bias}=-0.35$ (V) (solid curve), the photocurrent spectrum was extended to a longer cutoff wavelength of 2.6 µm.

The photocurrent spectra of the fabricated e-SWIR APD, referred to henceforth as the device, were measured using a Bruker INVENIO^®^ Fourier transform infrared (FTIR) spectrometer by Bruker Corporation at 40 Manning Rd, Billerica, MA, USA. The device replaced the internal DTGS detector of the FTIR, and the photocurrent signals were collected and transmitted to the FTIR through the equipment’s external A/D converter unit. The device was tested through top-illumination from the surface-normal direction. The device was not polished on the backside. The spot size of the FTIR illumination light was estimated to be 2 mm in diameter. Figure 4 displays the measured FTIR photocurrent spectra at different bias voltages. At a low bias voltage $V_{bias}=-0.12$ (V) (dashed curve), the photocurrent spectra were primarily below the cutoff wavelength of a typical GaSb photodetector at 1.72 µm. Conversely, at a slightly higher bias voltage of $V_{bias}=-0.35$ (V) (solid curve), the device exhibited a longer detection wavelength with a cutoff wavelength of $\lambda_{cutoff}=2.6\;\mu m$. This corresponded to the collection of the photocurrent generated in the In_0.3_Ga_0.7_As_0.25_Sb_0.75_ layer under the higher bias voltage. Note that the low-noise preamplifier used with the FTIR spectrometer had a low current overflow level, preventing the measurement of the photocurrent at higher biases. Nevertheless, the long cutoff wavelength of $\lambda_{cutoff}=2.6\;\mu m$ at a low bias voltage of $V_{bias}=-0.35$ (V) still demonstrated the APD’s capabilities in achieving the long cutoff wavelength of $\lambda_{cutoff}=2.6\;\mu m$ at RT.

Note that the photocurrent was nearly zero at a wavelength of 2.6 µm, with a bias of $V_{bias}=-0.35$ V. This occurred due to the electron band filling effect, also known as the Moss–Burstein effect [32,33,34], where the bias voltage shifts the Fermi-level and thus changes the occupations of the conduction bands and valence band. Similar phenomena have previously been reported and analyzed [35].

**Figure 5:**
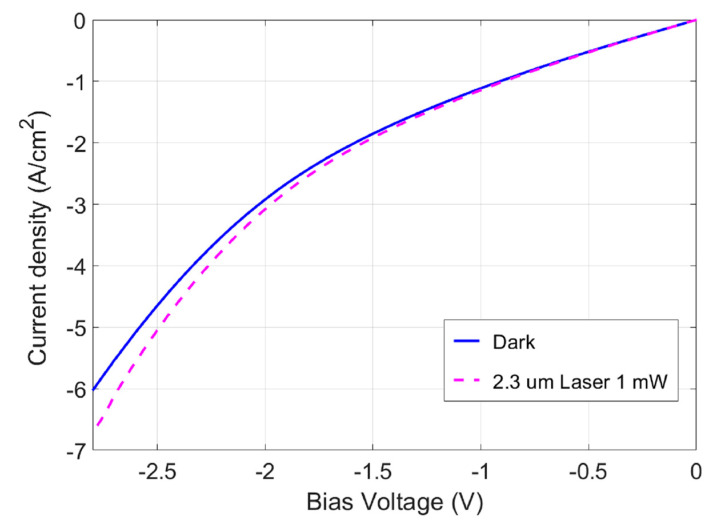
Measured I–V curve (dashed curve) of the APD under 2.3 µm laser illumination compared to the dark I–V curve (solid curve).

To obtain the photoresponse at higher bias voltages, the current versus bias voltage characteristics (i.e., I–V curves) of the APD were measured under the illumination of a distributed feedback (DFB) semiconductor laser (Eblana Photonics^®^ EP2327) by Eblana Photonics at 3 West Pier Campus, Dun Laoghaire, Co. Dublin, A96 A621, Ireland with an emission wavelength of $\lambda_{laser}=2327\;nm$ (referred to henceforth as the 2.3 µm laser) from the top illumination. Figure 5 shows the measured I–V curve under laser illumination (dashed curve) compared to the dark I–V curve (solid curve) without laser illumination. The APD-received laser power was estimated to be $P_{r}=41\;\mu W$. The photocurrent under 2.3 µm laser illumination confirmed the APD’s ability to detect IR light wavelengths exceeding 2.0 µm. Additionally, the steep slopes of the I–V curves at higher bias voltages than $V_{bias}<-1.5$ (V) indicated the avalanche gain-induced high current. The avalanche process at the low bias voltage was attributed to the stepwise heterojunction design [29], which reduced the depletion width and thus led to a low breakdown voltage. It may have also been due to the breakdown at the sharp edges of the device.

**Figure 6:**
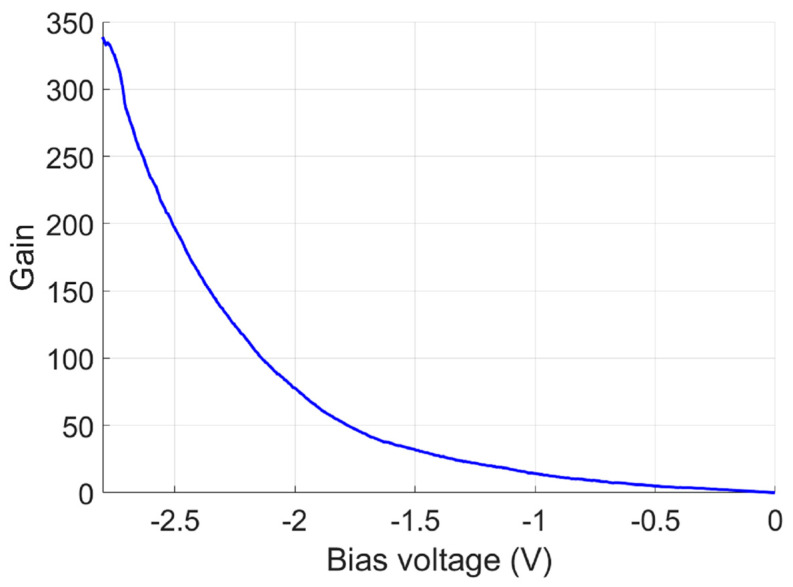
Calculated avalanche gain of the device under different voltage biases.

To obtain the avalanche gains and the excess noise factor, we analyzed the noise components and the measured noise of the APD under different biases. The thermal noise current spectral density $i_{th}/\sqrt{B}$ in $A/\sqrt{Hz}$ can be expressed as:

(1) $$i_{th}/\sqrt{B}=\sqrt{\frac{4k_{B}T}{R_{d}}}$$

where $k_{B}$ is the Boltzmann’s constant, T is the absolution temperature in kelvin (°K), and $R_{d}$ is the differential resistance of the APD, which can be calculated from the I-V characteristics shown in Figure 3. The shot noise current spectral density $i_{sh}/\sqrt{B}$ in $A/\sqrt{Hz}$ can be written as [36]:

(2) $$i_{sh}/\sqrt{B}=\sqrt{2qM^{2}{\left(I_{ph}+I_{d}\right)|}_{0}F(M)}$$

where M is the avalanche gain; $q=1.6\times 10^{-19}$ (C) is the amount of charge of an electron; and the ${\left(I_{ph}+I_{d}\right)|}_{0}$ term is the sum of the photocurrent ($I_{ph}$) and dark current ($I_{d}$) before avalanche. F(M) is the excess noise factor, which can be expressed as follows for the hole-dominated impact ionization process [36]:

(3) $$F\left(M\right)=kM+(1-k)M{\left(\frac{M-1}{M}\right)}^{2}$$

where k=β/α represents the ratio of the impact ionization coefficient for holes, β, to that for electrons, α. The thermal noise current spectral density $i_{th}/\sqrt{B}$ was calculated to be $\sim 2\times 10^{-11}A/\sqrt{Hz}$, whereas the shot noise current spectral density was > $\sim 1\times 10^{-10}A/\sqrt{Hz}$, even at the avalanche gain of M=1. This indicates that the APD was shot-noise-dominated. The avalanche multiplication gain M was calculated using the following equation:

(4) $$M=\frac{{\left(I_{ph}-I_{d}\right)|}_{Avalanche}}{{\left(I_{ph}-I_{d}\right)|}_{0}}$$

where ${\left(I_{ph}-I_{d}\right)|}_{Avalanche}$ is the avalanched APD photocurrent and ${\left(I_{ph}-I_{d}\right)|}_{0}$ is the photocurrent before the avalanche. Figure 6 shows the avalanche gain M at different bias voltages. A high gain of M~190 was achieved at the bias voltage of $V_{bias}=-2.5$ (V).

The temporal response was determined by the RC constant of the e-SWIR APD, which was calculated to be ~ 40 nanoseconds (ns).

**Figure 7:**
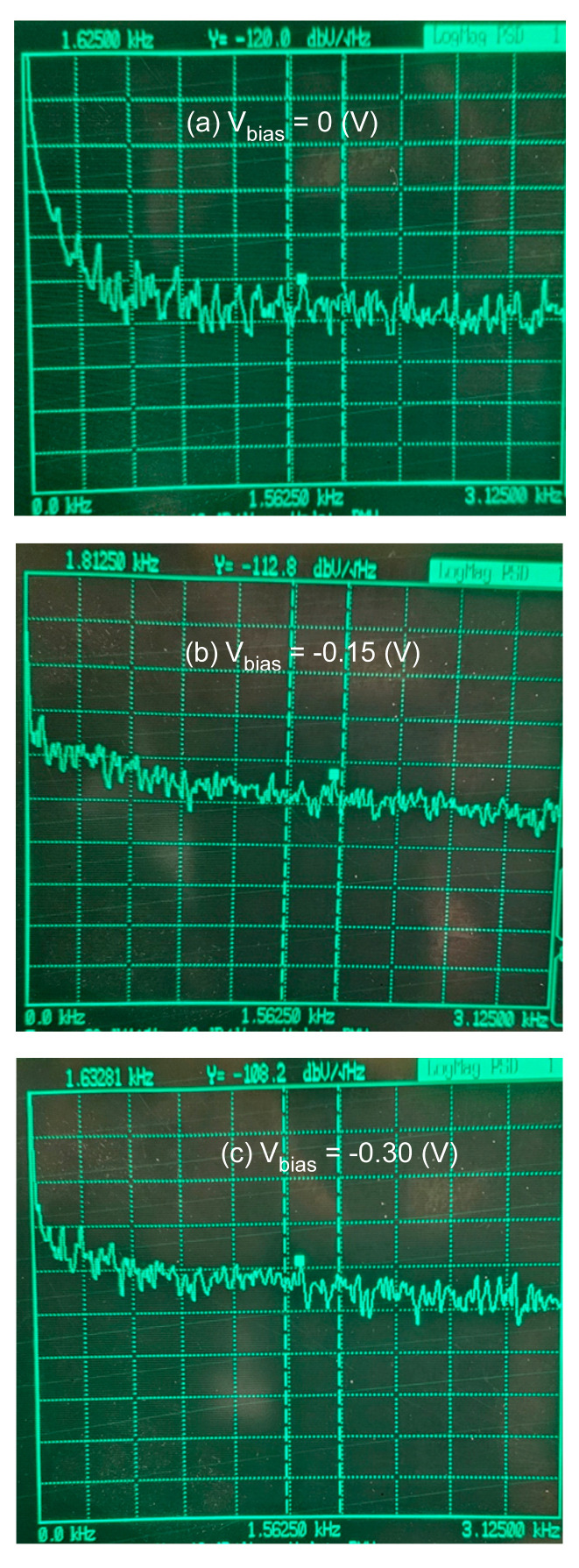
Measured VSD of the APD under different voltage biases.

The noise spectral density ${VSD}_{dBV}$ in $dBV/\sqrt{Hz}$ of the device was measured using an SR770 FFT spectrum analyzer from Stanford Research Systems at 1290-D Reamwood Ave. Sunnyvale, CA, USA after converting the current signal to a voltage signal through a low-noise transimpedance preamplifier. Figure 7a, Figure 7b, and Figure 7c show the measured VSD at the bias voltages of 0 (V), −0.15 (V), and −0.30 (V), respectively. The measured VSD were −$120.0\;dBV/\sqrt{Hz}$, −$112.8\;dBV/\sqrt{Hz}$, and −$108.2\;dBV/\sqrt{Hz}$, respectively. The noise VSD of −$120.0\;dBV/\sqrt{Hz}$ at the bias voltage of $V_{bias}=0$ (V) included the noise floor of the measurement equipment and was, thus, higher than the real VSD of the device.

The VSD in $dBV/\sqrt{Hz}$ was related to the noise spectral density $i_{sh}/\sqrt{B}$ by:

(5) $${VSD}_{dBV}=20{log}_{10}\left(\frac{i_{sh}S/\sqrt{B}}{1V/\sqrt{B}}\right)$$

**Figure 8:**
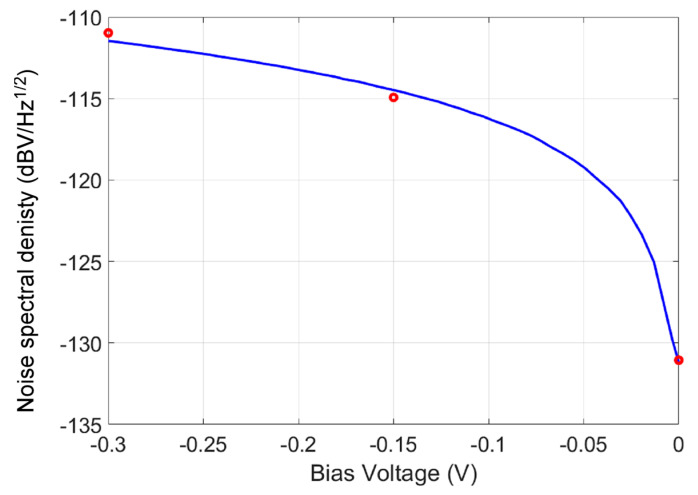
Calculated VSD values at different bias voltages (solid curve). The circles are the measured VSD values after noise floor adjustment.

Combining Equations (2)–(5), it was possible to calculate the VSD at different bias voltages and obtain the k factor value through curve-fitting with the measured VSD values. Figure 7 shows the calculated VSD at different bias voltages. The measured VSD values are marked in Figure 8 using circles. Note that the measured VSD values were adjusted by removing the noise floor of the equipment. The k factor value was determined to k=0.003 by curve fitting.

**Figure 9:**
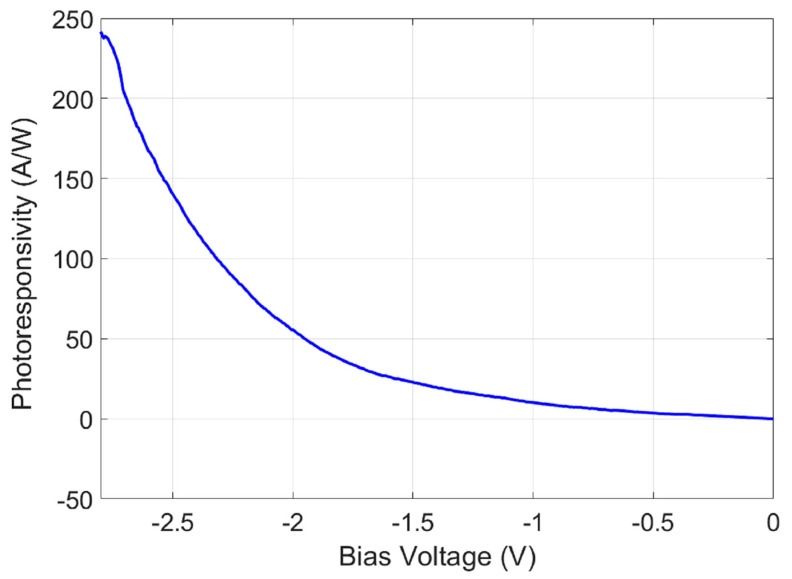
Photoresponsivity R at different voltage biases.

The photoresponsivity R was defined as [37]:

(6) $$R=\frac{I_{ph}}{P_{r}}$$

where $I_{ph}$ is the photocurrent and $P_{r}$ is the laser power received by the device. Figure 9 shows the photoresponsivity R at different bias voltages.

The noise equivalent power (NEP) in $W/\sqrt{Hz}$ was defined as [37]:

(7) $$NEP=\frac{i_{noise}}{R}$$

**Figure 10:**
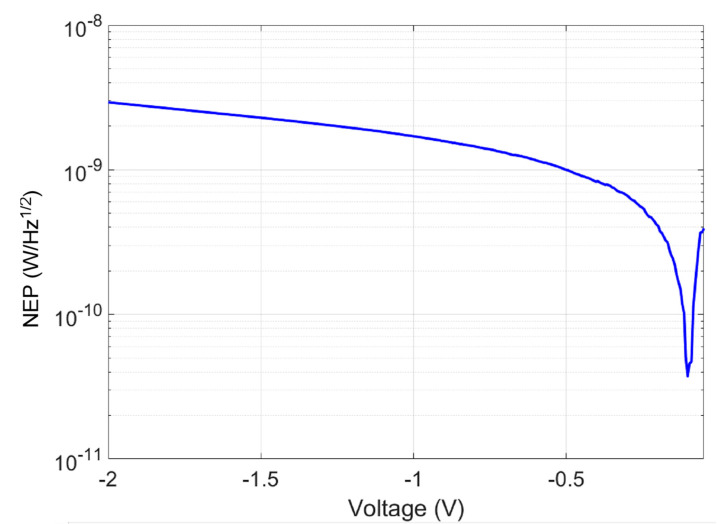
Noise equivalent power (NEP) $W/\sqrt{Hz}$ at different biases.

$i_{noise}$ is the calculated current noise using the k factor value of k=0.003. Figure 10 shows the NEP at different bias voltages.

**Figure 11:**
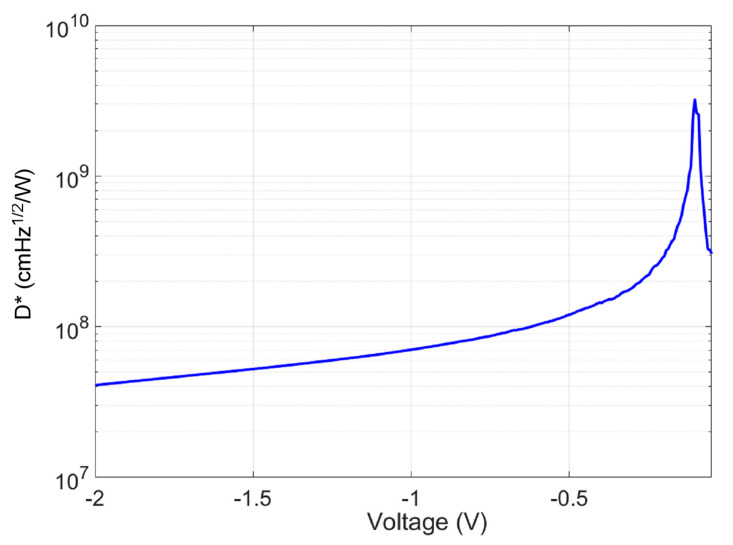
D* ($cmW/\sqrt{Hz}$) at different biases.

The specific photodetectivity (D*) in $cm\sqrt{Hz}/W$ was defined as [37]:

(8) $$D^{*}=\frac{\sqrt{A}}{NEP}$$

where A is the area of the APD. Figure 11 shows the D* at different biases. The reverse bias increases caused a rise in noise level, resulting in a decrease in D*.

## 4. Conclusions

In conclusion, herein, an RT e-SWIR APD with a cutoff wavelength of 2.6 µm is demonstrated based on the In_0.3_Ga_0.7_As_0.25_Sb_0.75_/GaSb heterostructures lattice-matched on GaSb substrates with high material quality. The e-SWIR APD shows a high multiplication gain of M~190 at a low bias of $V_{bias}=-2.5\;V$. The high internal gain enabled highly sensitive e-SWIR photodetection. The capability for RT operation eliminated the need for cooling, thus facilitating remote chemical sensing and infrared (IR) imaging with significantly reduced SWaP.

## Figures and Tables

**Table 1:** Comparison of this e-SWIR APD with previously reported APDs.

Performance Features	This Work	AlInAsSb/GaSb APD [17]	Ge_1−x_Sn_x_ APD [24]	Stepwise WSe_2_ APD [29]
Cutoff Wavelength (µm)	2.6	2.0	2.003	<1
Gain	190	>100	>15	>100
Operating Temperature	RT	RT	RT	RT
Reverse Bias (V)	<2.5	>20	<10	<1.6
K-Factor	k~0.003	k~0.01	N/A	N/A
Photoresponsivity (A/W)	>140	N/A	0.33	N/A
$\mathrm{Photodetectivity}\;D*\;(cm\sqrt{Hz}/W)$	$3\times 10^{7}$	N/A	N/A	N/A

N/A: Not reported in the reference.

## Data Availability

The raw data supporting the conclusions of this article will be made available by the authors on request.
